# Supplementary material for: Tissue Forge: Interactive biological and biophysics simulation environment
Source: PLoS Comput Biol. 2023 Oct 23;19(10):e1010768. doi: 10.1371/journal.pcbi.1010768 (PMC10621971; doi:10.1371/journal.pcbi.1010768)
Supplement: S1 File — Instructions for installing pre-built Tissue Forge binaries. (PDF) [file pcbi.1010768.s001.pdf]

Installing Tissue Forge

May 6, 2023

Additional documentation and the GitHub repository for Tissue Forge are available at:

- Tissue Forge GitHub repository:
  `https://github.com/tissue-forge/tissue-forge/`
- Tissue Forge Documentation:
  `https://tissue-forge-documentation.readthedocs.io/en/latest/`
- Tissue Forge Python API Documentation:
  `https://tissue-forge-python-api-documentation.readthedocs.io/en/latest/`

Binary distributions of Tissue Forge v0.1.1 are available for 64-bit Windows, MacOS and Linux and Python versions 3.7-3.9 via conda from the *tissue-forge* channel. In a terminal with the conda command available, Tissue Forge can be installed with a single command,

```
conda install -c conda-forge -c tissue-forge tissue-forge
```

Users are recommended to install Tissue Forge into a new environment or existing environment that is not the base environment of their conda installation. After installation into a conda environment, Tissue Forge is available upon activation of the environment in the typical way. Users can utilize built-in Tissue Forge support for Jupyter Notebook by also installing the *notebook*, *ipywidgets* and *ipyevents* packages.

```
conda install -c conda-forge notebook ipywidgets ipyevents
```
