# Supplementary figures and images for: Tissue Forge: Interactive biological and biophysics simulation environment
Source: PLoS Comput Biol. 2023 Oct 23;19(10):e1010768. doi: 10.1371/journal.pcbi.1010768 (PMC10621971; doi:10.1371/journal.pcbi.1010768)

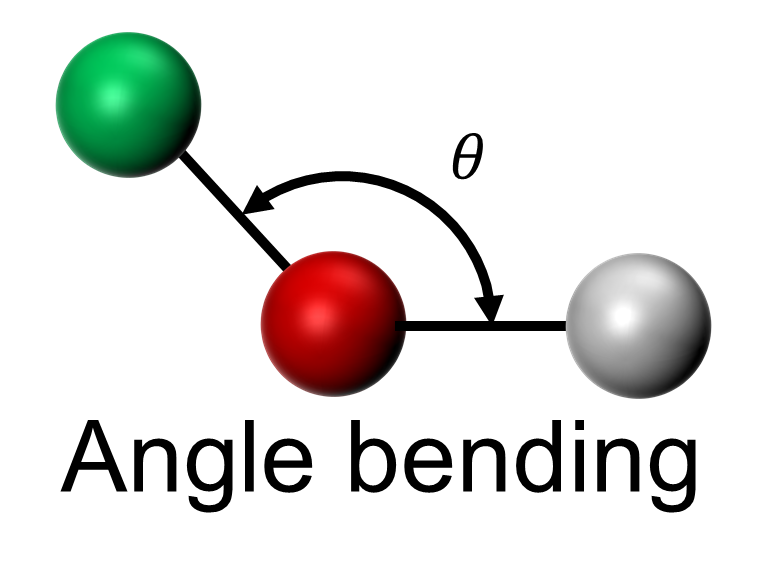

Supplement: S8 File — Tissue Forge version 0.1.1 source code. (ZIP) [file pcbi.1010768.s008.zip › tissue-forge-main/docs/main/source/angle.png]

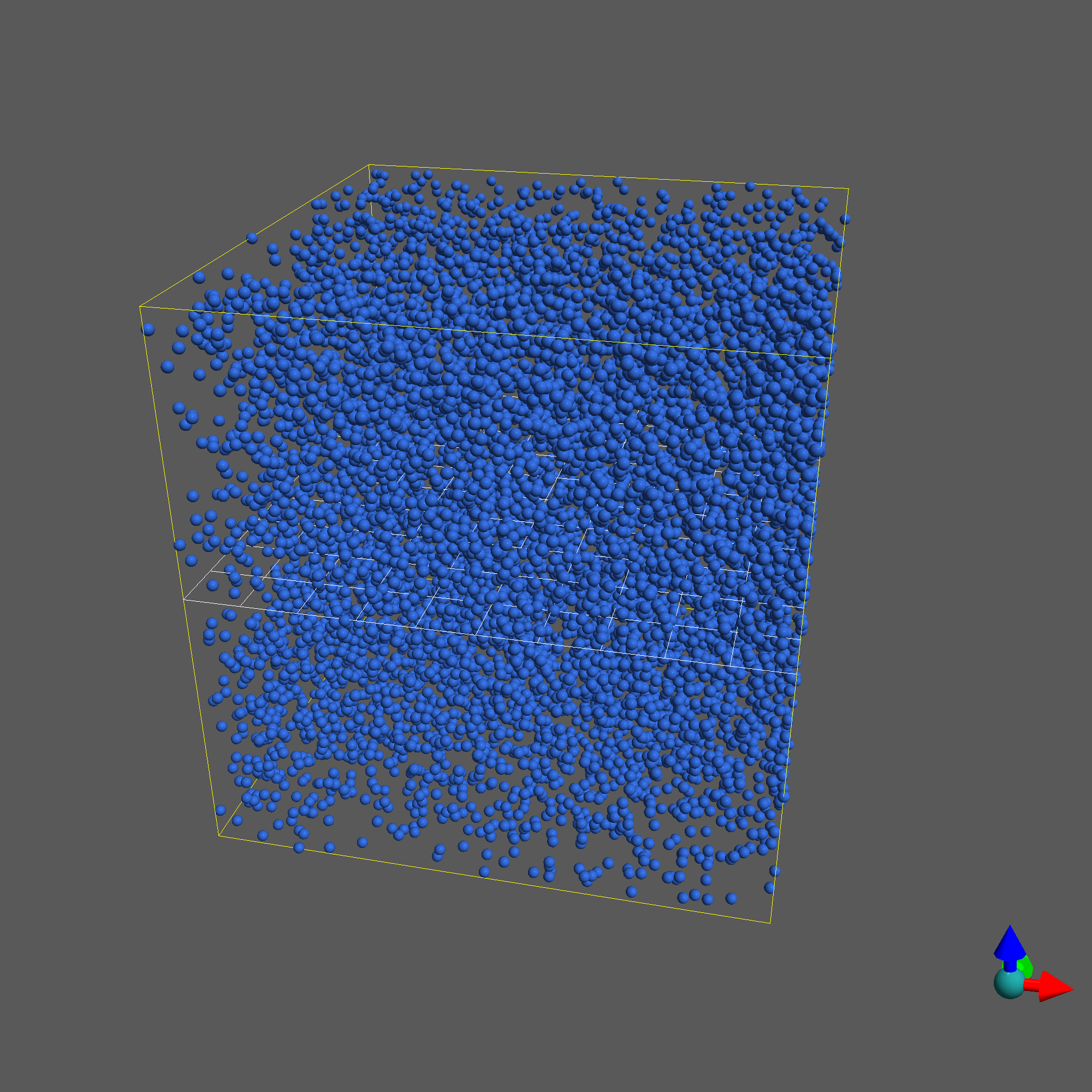

Supplement: S8 File — Tissue Forge version 0.1.1 source code. (ZIP) [file pcbi.1010768.s008.zip › tissue-forge-main/docs/main/source/argon.png]

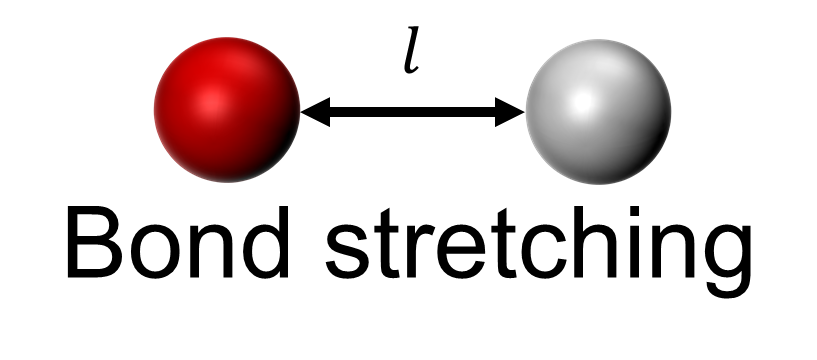

Supplement: S8 File — Tissue Forge version 0.1.1 source code. (ZIP) [file pcbi.1010768.s008.zip › tissue-forge-main/docs/main/source/bond.png]

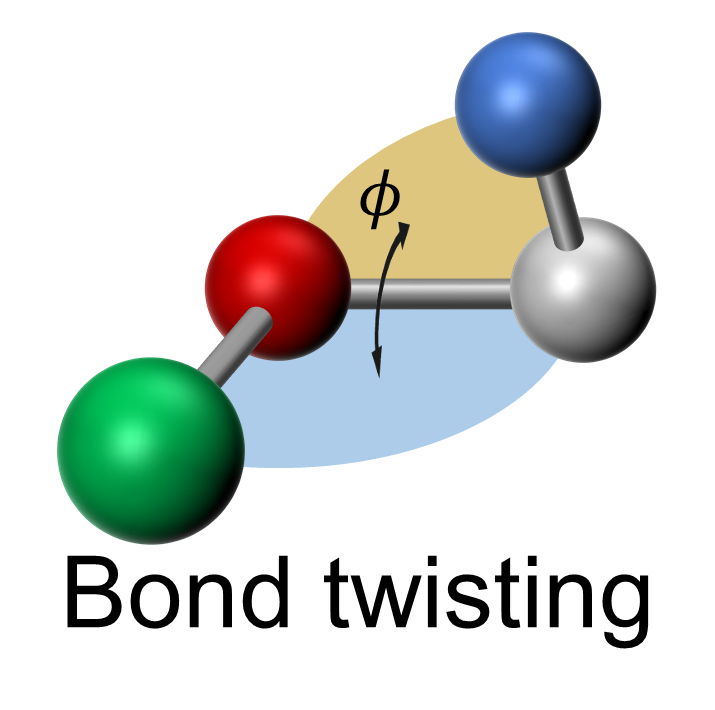

Supplement: S8 File — Tissue Forge version 0.1.1 source code. (ZIP) [file pcbi.1010768.s008.zip › tissue-forge-main/docs/main/source/dihedral.png]

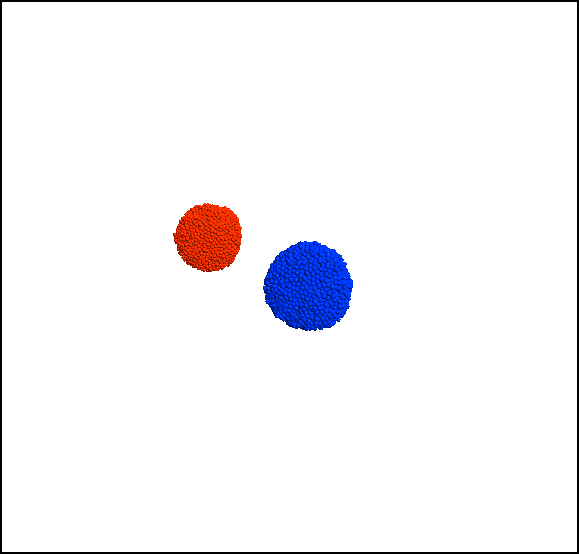

Supplement: S8 File — Tissue Forge version 0.1.1 source code. (ZIP) [file pcbi.1010768.s008.zip › tissue-forge-main/docs/main/source/droplets_3d.gif]

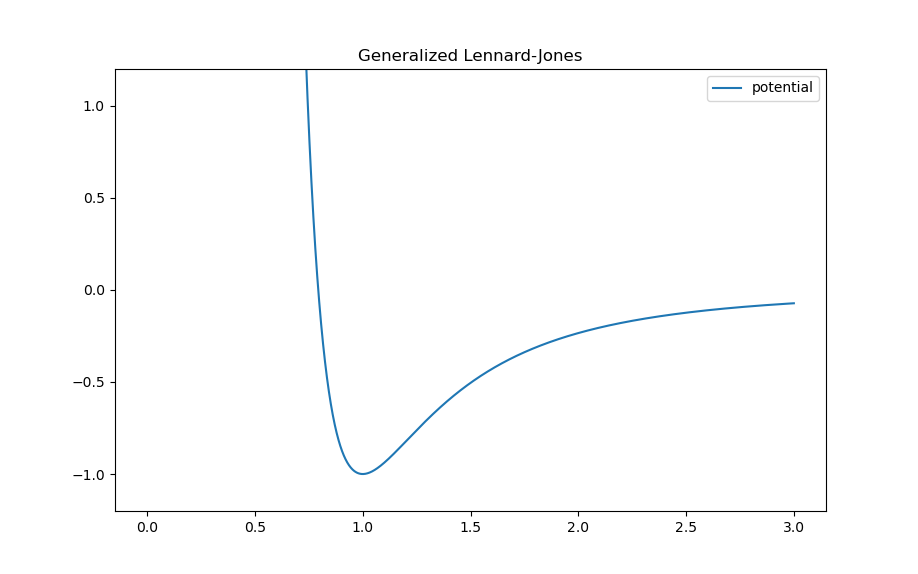

Supplement: S8 File — Tissue Forge version 0.1.1 source code. (ZIP) [file pcbi.1010768.s008.zip › tissue-forge-main/docs/main/source/glj_plot.png]

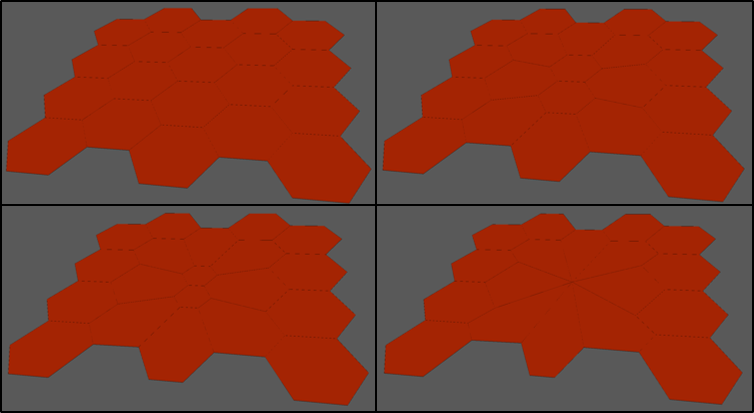

Supplement: S8 File — Tissue Forge version 0.1.1 source code. (ZIP) [file pcbi.1010768.s008.zip › tissue-forge-main/docs/main/source/models/vertex/solver/bind_example1.png]

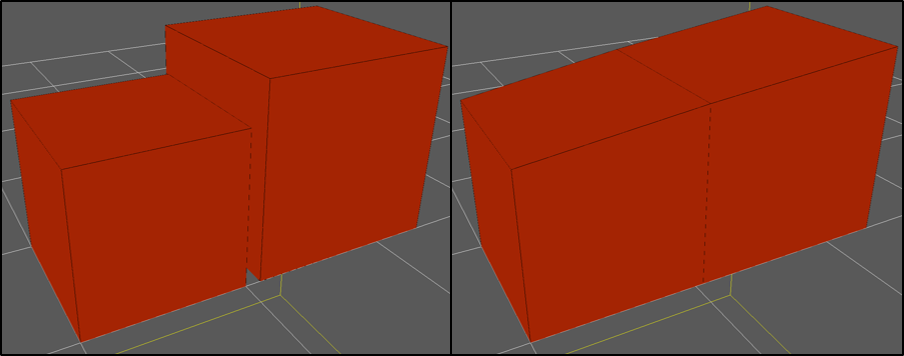

Supplement: S8 File — Tissue Forge version 0.1.1 source code. (ZIP) [file pcbi.1010768.s008.zip › tissue-forge-main/docs/main/source/models/vertex/solver/body_example1.png]

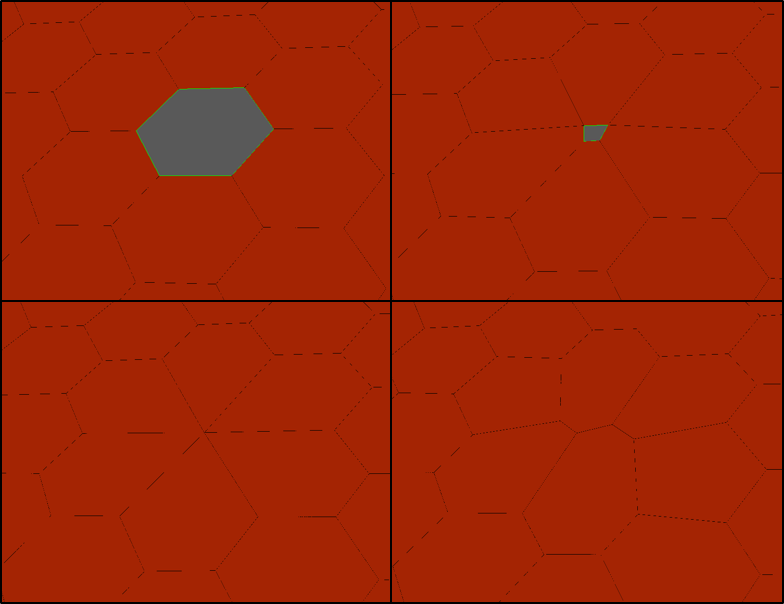

Supplement: S8 File — Tissue Forge version 0.1.1 source code. (ZIP) [file pcbi.1010768.s008.zip › tissue-forge-main/docs/main/source/models/vertex/solver/bond_example1.png]

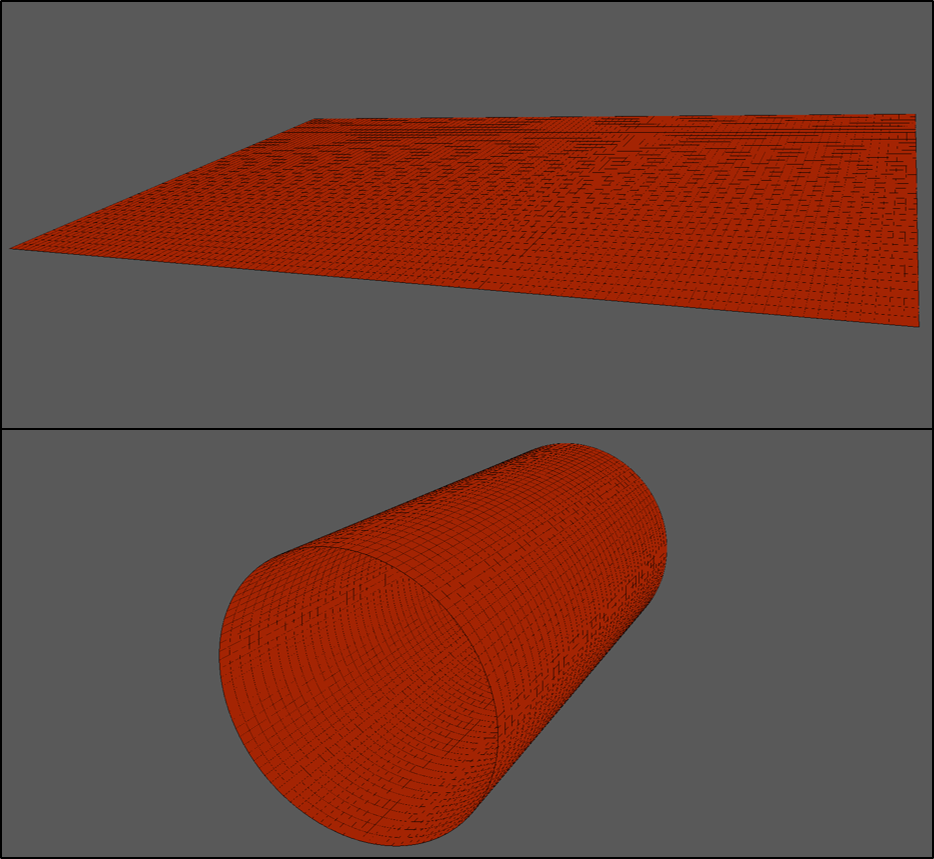

Supplement: S8 File — Tissue Forge version 0.1.1 source code. (ZIP) [file pcbi.1010768.s008.zip › tissue-forge-main/docs/main/source/models/vertex/solver/generator_example1.png]

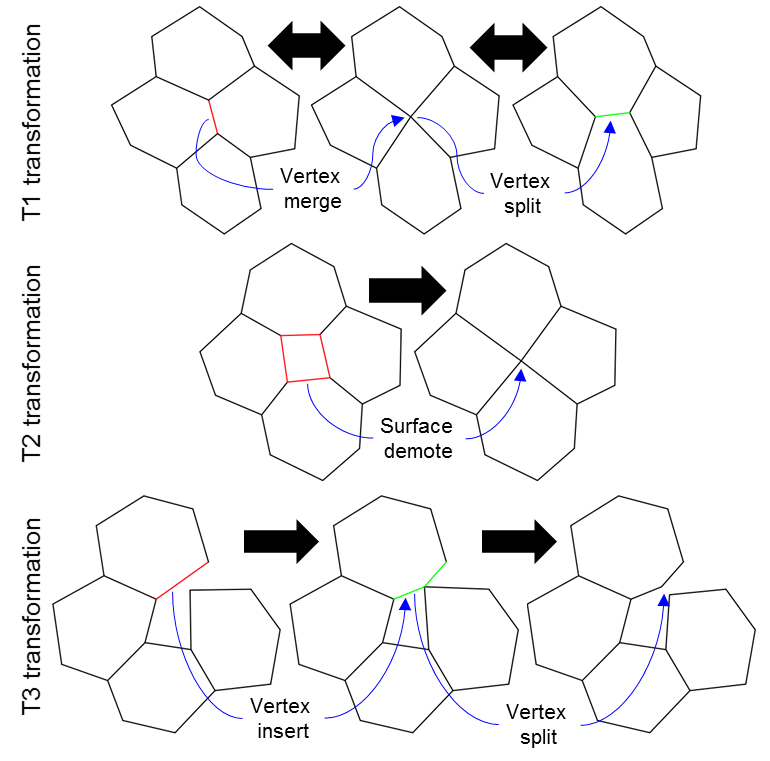

Supplement: S8 File — Tissue Forge version 0.1.1 source code. (ZIP) [file pcbi.1010768.s008.zip › tissue-forge-main/docs/main/source/models/vertex/solver/mesh_quality_2D.png]

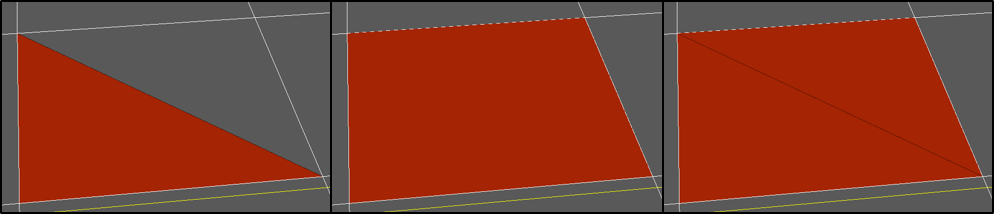

Supplement: S8 File — Tissue Forge version 0.1.1 source code. (ZIP) [file pcbi.1010768.s008.zip › tissue-forge-main/docs/main/source/models/vertex/solver/surface_example1.png]

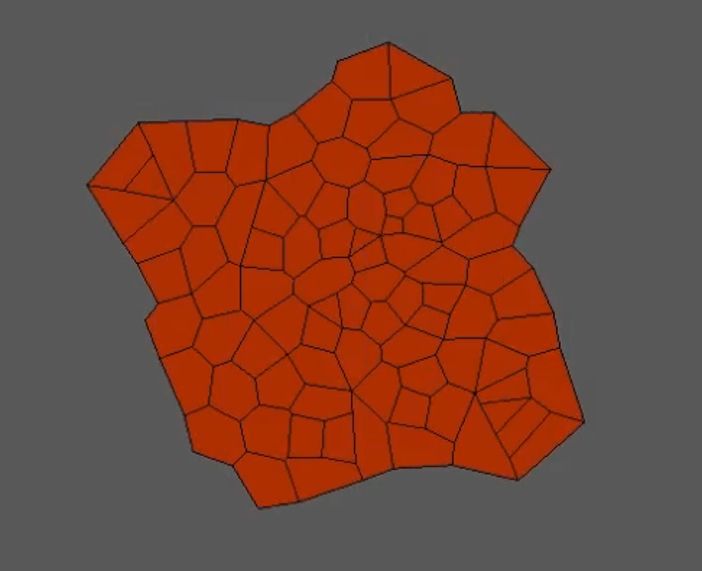

Supplement: S8 File — Tissue Forge version 0.1.1 source code. (ZIP) [file pcbi.1010768.s008.zip › tissue-forge-main/docs/main/source/models/vertex/solver/tf_interactive_2dsplitting.png]

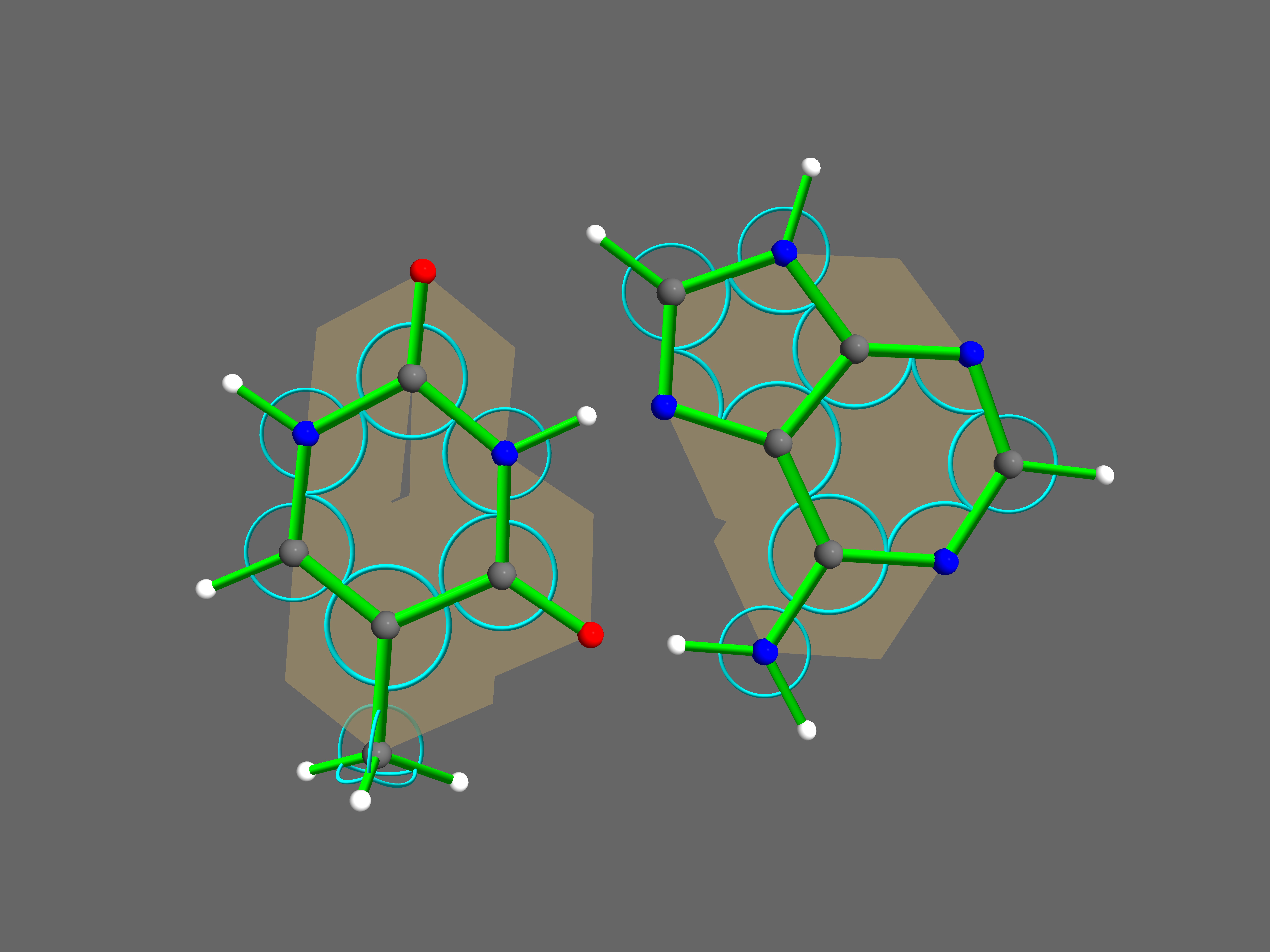

Supplement: S8 File — Tissue Forge version 0.1.1 source code. (ZIP) [file pcbi.1010768.s008.zip › tissue-forge-main/docs/main/source/nucleos_ta.png]
